# Genome characterization and population genetic structure of the zoonotic pathogen, *Streptococcus canis*

**DOI:** 10.1186/1471-2180-12-293

**Published:** 2012-12-18

**Authors:** Vincent P Richards, Ruth N Zadoks, Paulina D Pavinski Bitar, Tristan Lefébure, Ping Lang, Brenda Werner, Linda Tikofsky, Paolo Moroni, Michael J Stanhope

**Affiliations:** 1 Department of Population Medicine and Diagnostic Sciences, College of Veterinary Medicine, Cornell University, Ithaca, NY, 14853, USA; 2 Quality Milk Production Services, College of Veterinary Medicine, Cornell University, Ithaca, NY, 14853, USA; 3 Università degli Studi di Milano, Department of Health, Animal Science and Food Safety, Via Celoria 10, 20133, Milan, Italy; 4 Current address: Moredun Research Institute, Pentlands Science Park, Bush Loan, Penicuik and Royal (Dick) School of Veterinary Studies, University of Edinburgh, Scotland, UK; 5 Current address: Université de Lyon, Université Lyon 1, Centre National de la Recherche Scientifique, Ecologie des Hydrosystèmes Naturels et Anthropisés, Villeurbanne, France; 6 Current address: Department of Plant Pathology & Plant-Microbe Biology, Cornell University, Ithaca, NY, 14853, USA; 7 Current address: 4055 McIntyre Road, Trumansburg, NY, 14886, USA

**Keywords:** *Streptococcus canis*, Comparative genomics, Pathogen, Zoonotic, Mastitis, Lateral gene transfer, Host adaptation, Bovine, Canine

## Abstract

**Background:**

*Streptococcus canis* is an important opportunistic pathogen of dogs and cats that can also infect a wide range of additional mammals including cows where it can cause mastitis. It is also an emerging human pathogen.

**Results:**

Here we provide characterization of the first genome sequence for this species, strain FSL S3-227 (milk isolate from a cow with an intra-mammary infection). A diverse array of putative virulence factors was encoded by the *S. canis* FSL S3-227 genome. Approximately 75% of these gene sequences were homologous to known Streptococcal virulence factors involved in invasion, evasion, and colonization. Present in the genome are multiple potentially mobile genetic elements (MGEs) [plasmid, phage, integrative conjugative element (ICE)] and comparison to other species provided convincing evidence for lateral gene transfer (LGT) between *S. canis* and two additional bovine mastitis causing pathogens (*Streptococcus agalactiae*, and *Streptococcus dysgalactiae* subsp. *dysgalactiae*), with this transfer possibly contributing to host adaptation. Population structure among isolates obtained from Europe and USA [bovine = 56, canine = 26, and feline = 1] was explored. Ribotyping of all isolates and multi locus sequence typing (MLST) of a subset of the isolates (*n* = 45) detected significant differentiation between bovine and canine isolates (Fisher exact test: *P* = 0.0000 [ribotypes], *P* = 0.0030 [sequence types]), suggesting possible host adaptation of some genotypes. Concurrently, the ancestral clonal complex (54% of isolates) occurred in many tissue types, all hosts, and all geographic locations suggesting the possibility of a wide and diverse niche.

**Conclusion:**

This study provides evidence highlighting the importance of LGT in the evolution of the bacteria *S. canis*, specifically, its possible role in host adaptation and acquisition of virulence factors. Furthermore, recent LGT detected between *S. canis* and human bacteria (*Streptococcus urinalis*) is cause for concern, as it highlights the possibility for continued acquisition of human virulence factors for this emerging zoonotic pathogen.

## Background

Originally described as β-hemolytic streptococci isolated from dogs and cows that possessed the Lancefield group G antigen [1], *Streptococcus canis* has subsequently been isolated from a variety of animal sources including cats, rats, rabbits, minks, foxes, a Japanese raccoon dog, and humans [2-4]. The species is an important opportunistic pathogen of cats and dogs infecting a wide range of tissues such as the central nervous system, respiratory tract, genitourinary system, blood, skin, bones, cardiovascular system, and abdomen [1,4-6]. Infection can cause serious invasive disease, such as streptococcal toxic shock syndrome (STSS), necrotizing fasciitis (NF), septicemia, pneumonia, and meningitis, with numerous reports of fatal infection [5,7-9], whereas in cows *S. canis* can cause mastitis [10-12]. Of concern are the accumulating reports of human infection (including numerous cases of dog to human transmission) [13-16], with clinical manifestations similar to those seen in cats and dogs. For example, descriptions of human cases include soft tissue infection, bacteremia, urinary infection, bone infection, pneumonia, and two reports of death from sepsis [13].

Although the phylogeny of the species is not completely resolved, a general consensus from the literature shows *S. canis* to be closely related to *Streptococcus dysgalactiae* subsp. *dysgalactiae*, *Streptococcus dysgalactiae* subsp. *equisimilis*, and *Streptococcus pyogenes*[2,17-21]. *S. canis* and *S. dysgalactiae* subsp. *equisimilis* are both β-hemolytic streptococci that share the same Lancefield group G antigen. Consequently, by the Lancefield system they are indistinguishable, and have traditionally only been classified as group G streptococci (GGS) from either animal (*S. canis*) or human (*S. dysgalactiae* subsp. *equisimilis*) origin. Therefore, it’s possible that human *S. canis* infection has been underestimated [13,15]. Investigating this problem, Broyles *et al*. [22] performed a survey of human invasive infection using techniques capable of distinguishing *S. canis* from *S. dysgalactiae* subsp. *equisimilis*. Results showed a low frequency of *S. canis* in blood samples. However, their study was biased towards the characterization of isolates from blood samples (isolates from other body sites were less likely to be characterized).

In humans, STSS and NF are serious diseases typically caused by *S. pyogenes* infection. The emergence of strikingly similar STSS and NF in cats and dogs coupled with the close relationship between the causal species prompted preliminary investigation and subsequent discovery of two shared virulence factors between these species [23]. To shed light on the molecular basis of *S. canis* virulence and further investigate the role *S. pyogenes* and other species of *Streptococcus* may have played in its evolution we determined the first genome sequence for this pathogen and compared it to an extensive range of streptococcal genomes (40 species, 213 strains). In addition, we explored population structure among canine, feline, and bovine isolates.

Our findings reveal a diverse array of genes within the *S. canis* genome homologous to known virulence factors, including several established virulence factors from *S. pyogenes*, *Streptococcus agalactiae*, and *Streptococcus pneumoniae*. We found evidence for multiple LGT events between *S. canis* and (i) other bovine mastitis causing pathogens, and (ii) the human pathogen *Streptococcus urinalis*, suggesting LGT in both shared bovine and human environments. This LGT was mediated by a variety of mobile genetic elements [plasmid, phage, integrative conjugative element] that carried many of the virulence factors, highlighting the importance of LGT in the evolution of this pathogen and the potential for its emergence as a zoonotic pathogen.

## Result and discussion

### Assembly and general features of the genome

**Figure 1:**
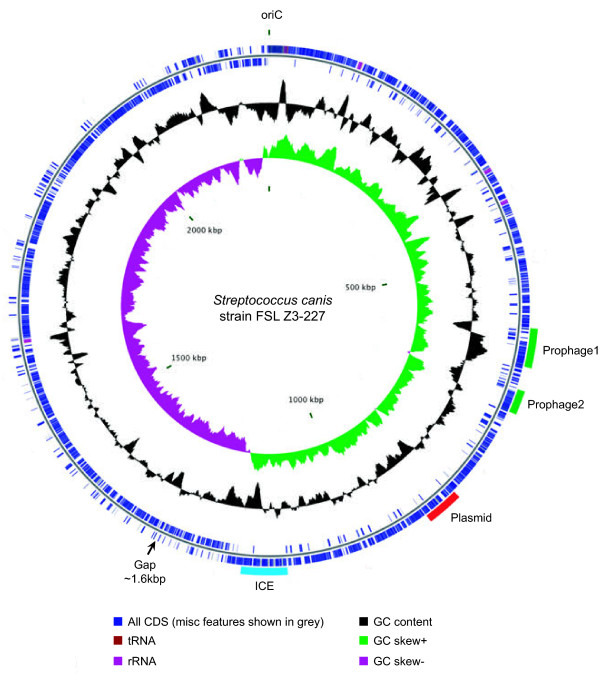
**Genome map of *Streptococcus canis *strain FSL Z3-227.** Starting from the outermost ring and moving inwards, rings show the location of: (1) four mobile genetic elements (see text for detailed description), (2) all annotated CDS on the leading strand, and (3) all annotated CDS on the lagging strand. Two innermost rings show GC content and GC skew. Map was created using the software CGView [26].

Roche/454 pyrosequencing produced 128,749 single-end reads and 140,788 paired-end reads that were assembled into 91 contigs (>200 bp) and eight scaffolds, representing an average 23X site coverage. Utilizing additional Illumina/Sanger sequencing and alignment to an optical map, the eight scaffolds were assembled into a single 2,267,856 bp contig. Unfortunately, we were unable to obtain sequence for one small section of the genome (Figure 1). The gap was within a collagen-like surface protein. The best BLAST hit at the NCBI nr database for each gene fragment (SCAZ3_06900 and SCAZ3_06785) was to an identically annotated gene within *S. agalactiae* (A909), (each fragment shared approximately 75% sequence identity). Alignment of the *S. canis* fragments to this gene suggested that we were missing approximately 1.6 kb. For *S. agalactiae* (A909), this gene contained 75 repeats of a 9 bp imperfect repeat (DCCRTCTTT). The majority of these repeats (70) were contained within a 2.5 kb region that spanned the *S. canis* gap and flanking regions. *S. canis* contained 26 repeats in the regions that flanked the gap. Consequently it seems likely that these repeats were also present within the un-sequenced section of the collagen gene for *S. canis* and that their presence confounded our sequencing attempts. Inclusion of this small gap made the total length of the genome approximately 2,269,456 bp. In comparison to 53 genome sequences representing 19 additional *Streptococcus* species, the *S. canis* genome was among the largest with regard to sequence length, ranking fourth (with one exception *S. agalactiae*-FSL S3-026], sequences were obtained from the manually curated RefSeq database at NCBI [see Additional file 1). *S. canis* had a relatively high number of CDS (2,212), ranking fifth, an intermediate number of tRNAs (67; range 41–80) and an average GC content of 39.7%. A 5,871 bp section of the genome appeared to have been perfectly duplicated (locus tags SCAZ3_r06686 through SCAZ3_t06810 plus 126 bp of non-coding DNA that preceded SCAZ3_r06686). The section contained an rRNA operon (16S-23S-5S) and 10 tRNAs that were immediately down stream (Val, Asp, Lys, Leu, Thr, Gly, Leu, Arg, and Pro). The entire section was perfectly duplicated immediately upstream (one nucleotide separated the two duplicated sections). Similar rRNA operon duplications are present in the genomes of *Streptococcus thermophilus* (LMD-9) and *Streptococcus salivarius* (CCHSS3). The number of rRNA operons in publicly available *Streptococcus* genomes ranges from one to seven, and the number within the *S. canis* genome was again relatively high, with six. It is possible that this reflects selection for rapid growth. For example, during rapid growth genes are likely to be expressed at high levels, and this is often associated with codon usage bias [24], which in turn, has been shown to be positively correlated with the number of rRNA operons within a bacterial genome [25].

### Virulence factors

A total of 291 CDS within the *S. canis* genome were homologous with established virulence factors in the Virulence Factor of Pathogenic Bacteria database (VFDB) (available at http://www.mgc.ac.cn/VFs/main.htm) (see Additional file 2). Throughout the manuscript, two genes (query and subject) are considered homologous if they can be locally aligned using BLAST with an *E* value of 1e-5 or less. A refined search, focusing only on established *Streptococcus* virulence factors, detected 34 CDS homologous to three other species of streptococci: *S. pyogenes* (17), *S. agalactiae* (9), and *S. pneumoniae* (8). Of these, the majority (50%) was homologous with *S. pyogenes*, likely reflecting the close relationship between these two species. More specifically, 9 of the 17 *S. pyogenes* virulence factors homologous to *S. canis* were categorized as either exoenzymes or complement proteases. These gene products damage tissue, and may contribute to necrotizing fasciitis. When considering all 291 of the virulence factors homologous to *S. canis*, there were only three additional genes with similar categorization, two of these homologous to *S. pneumoniae.* Consequently, it appears that several genes possibly involved in necrotizing fasciitis are shared between *S. canis* and *S. pyogenes*. In contrast, *S. canis* CDSs were not homologous with genes producing pyrogenic exotoxins associated with toxic shock syndrome. However, *S. canis* possessed two other streptoccocal toxin-producing genes: streptolysin O (SLO) (*S. pyogenes*) and CAMP factor (*S. agalactiae*) [27,28]. Two *S. canis* genes were homologous to a well-characterized *S. pyogenes* virulence factor, the M protein (emm18), which aids in antiphagocytosis, adherence, and cellular invasion [29]. However, unlike *S. pyogenes*, these genes were not located within a contiguous 35-gene pathogenicity island that is found in all currently genome sequenced strains of *S. pyogenes*[30]. A BLASTn search of the NCBI nr database showed SCAZ3_01465 to be homologous with the gene SPASc from *S. canis*[31] (accession number: FJ594772). Global nucleotide sequence alignment showed these sequences to have 87.7% identity. Yang *et al.*[31] showed experimentally that SPASc was a new protective antigen, however they did not report the strain ID or isolation source. For SCAZ3_11010, a BLASTn search of the NCBI nr database returned no hits. However, a BLASTp search returned numerous hits and the gene with the most sequence similarity was an emm-like cell surface protein CspZ.2 of *Streptococcus equi* subsp. *zooepidemicus* ATCC 35246 (31% identity, 48% coverage). Neither SCAZ3_01465 nor SCAZ3_11010 were homologous with the *S. canis emm* gene type stG1389 (accession number EU195120) reported from one human and two canine sources [22]. These findings confirm previous studies showing that some *S. canis* isolates can possess M like proteins [18,22,23] and additionally show that a diversity of M like proteins is possible for *S. canis* strains.

*S. canis* also possessed the nine gene *sag* operon (*sag*ABCDEFGHI) responsible for the production of streptolysin S (SLS) [32]. Both SLS and SLO are toxins that lyse mammalian erythrocytes [33], and the toxicity of SLS has been shown to contribute to necrotizing fasciitis [34,35]. Furthermore, it has been suggested that SLS interacts with numerous additional virulence factors to accelerate necrosis [36]. These factors include SLO, the M protein, and proteases. Genes for all these factors can be present in the *S. canis* genome. Utilizing a BLASTn search of the NCBI nr database and subsequent global nucleotide sequence alignment, we detected the *sag* operon in four additional *Streptococcus* species (strain and percent sequence identity with *S. canis* are given in parentheses): *S. dysgalactiae* subsp. *equisimilis* (ATCC 12394; 81.1%), *Streptococcus pseudoporcinus* (LQ940-04 T; 78.8%), *S. pyogenes* (MGAS10270; 76.5%), and *Streptococcus iniae* (9117; 74.4%). The likely presence of the *sag* operon in *S. dysgalactiae* subsp. *equisimilis* was first shown by Humar *et al*. [34] who detected a functional *sag*A homolog in strains capable of producing SLS. *S. canis* and *S. iniae* are somewhat distinctive in that the other species are predominately human pathogens, whereas the former are predominately animal pathogens (*S. iniae* is a common fish pathogen), although occasionally are associated with zoonotic disease [37-39]. *S. dysgalactiae* subsp. *dysgalactiae*, which is predominantly associated with disease in animals but not in humans, lacks an intact *sag* operon, possessing only *sag*A and *sag*I. The occurrence of the complete operon in the other close relatives of *S. canis* (*S. dysgalactiae* subsp. *equisimilis* and *S. pyogenes*) suggests that *S. dysgalactiae* subsp. *dysgalactiae* may have lost the remainder of the genes from the operon. However, the occurrence of the operon in two species more distantly related to *S. canis,* that are themselves likely not sister species (*S. pseudoporcinus* and *S. iniae*) [40], is suggestive in this case of lateral gene transfer of the operon. Fish handling and close association with domestic dogs may have facilitated lateral gene transfer between species occupying human and animal hosts [14,16,41].

### Genes specific to *S. canis* (FSL Z3-227)

To identify genes that are likely *S. canis* species specific from genes present in multiple species of the genus, we performed a clustering analysis among 214 *Streptococcus* genomes representing 41 species including *S. canis* (see Methods section and Additional file 3). The analysis identified 97 genes that were not homologous to any other gene in the analysis and were unique to *S. canis* (see Additional file 2). Unfortunately, all were annotated as hypothetical proteins, highlighting the need for future studies exploring functional genomics for this species. *S. canis* belongs to the pyogenic 16S rRNA phylogenetic group [42]. Limiting the comparison to pyogenic genomes (14 species and 40 genomes, excluding *S. canis*), we identified an additional 14 genes unique to the *S. canis* genome (see Additional file 2). Two of these genes were homologous to two established virulence factors in the VFDB. The first gene (neuraminidase C, SCAZ3_10275) was homologous with neuraminidase B (*nanB*) from *S. pneumoniae* (TIGR4). The product of *nanB* is a glycosidase that, by damaging surface glycans and exposing the cell surface, aids in the adhesion to host cells and is therefore likely important in host invasion [43]. The second gene (Gfo/Idh/MocA family oxidoreductase, SCAZ3_10270) was homologous with *bplA* from *Bordetella pertussis* (Tohama I). In *Bordetella*, *bpl* genes are involved in the synthesis of the LPS, which has been shown to be essential for the expression of complete virulence in mice [44]. Given that the additional 14 genes unique to the *S. canis* genome were absent in the other pyogenic genomes, it is possible that these loci were gained via LGT. The two genes homologous to the virulence factors discussed above, were contiguous in the genome suggesting they were gained in a single evolutionary event.

### Integrative plasmid

**Figure 2:**
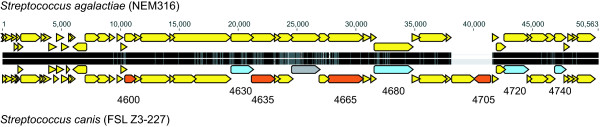
**Gene organization within putative integrative plasmids for *S. agalactiae *strain NEM316 (plasmid designated pNEM316-1) and *S. canis *strain FSL Z3-227 (plasmid designated FSL Z3-227-p).** Locus IDs for (i) CDS with putative plasmid functional role (blue arrows), and (ii) CDS homologous with established virulence factors (red arrows) are shown for *S. canis* (see text for detailed description). Grey arrow shows a miscellaneous feature that is a common BLAST hit with the M protein from *S. pyogenes*. Two horizontal black/grey bars are a generalized representation of the aligned nucleotide sequences, with black shading representing 100% identity. Figure created using Geneious v5.1.2 and Adobe Illustrator.

With the exception of two loci, *S. canis* shared a contiguous section of 53 CDS with *S. agalactiae* (NEM316) (Figure 2) (see also Additional file 2: locus tags SCAZ3_04485 through SCAZ3_04760 [50,114 bp]). Sequence identity between the shared 53 CDS was very high: 99.2%. First described in *S. agalactiae* (NEM316) [45], this section of DNA (designated pNEM316-1) was proposed to be a putative integrative plasmid (it could exist in circular form and was present as three copies within the genome). Here we designate the *S. canis* copy of the putative plasmid as FSL Z3-227-p. The last 24 bp at the terminal ends of pNEM316-1 were imperfect repeats of themselves (see Additional file 4). Alignment of pNEM316-1 with FSL Z3-227-p revealed identical terminal sequence for FSL Z3-227-p. Putative recombination *attL* and *attR* sites were also identified. As for pNEM316-1, these sites were 9 bp direct repeats.

Annotation of several *S. canis* CDS within this 50 kb region suggest a plasmid functional role (Figure 2 and Additional file 2). For example, DNA topoisomerase (SCAZ3_04630), conjugation protein (SCAZ3_04680, SCAZ3_04720), and plasmid partition protein (SCAZ3_04740) were identified. In addition, four CDS were homologous with established virulence factors (see Additional file 2, locus tags are highlighted in red in the annotations worksheet). Specifically, SCAZ3_04635 (ATP-dependent *clp* protease) was homologous with *clpE*, an ATP-dependent protease from *Listeria monocytogenes*; *clp* genes have been shown to play a role in competence, development, and stress survival (thermotolerance) in *S. pneumoniae*[46]. SCAZ3_04705 (DNA-cytosine methyltransferase) was homologous with a putative DNA methylase from *Escherichia coli* (strain 536), which is located within a pathogenicity island [47] that included MGEs as an integral part of its evolutionary history [48]. Likewise, SCAZ3_04705 is located within a MGE and its specific function may involve plasmid defense. For example, the conjugative plasmid Tn5252, which infects streptococci, contains DNA methyltransferases that may methylate the plasmid DNA, thereby providing protection from host restriction nucleases [49]. SCAZ3_04600 (DNA-entry nuclease) was homologous with a putative deoxyribonuclease (DNase) from *S. pyogenes.* DNA-entry nuclease facilitates entry of DNA into competent bacterial cells and may aid plasmid cell-to-cell transmission [50]. Although the role of DNase in *S. pyogenes* is not fully understood, Sumby *et al*. [51] provided strong evidence that it may enhance host evasion. SCAZ3_04665 (cell wall surface anchor family protein) was homologous with a gene from *Enterococcus faecalis* producing a putative aggregation substance that was categorized as an adherence factor. SCAZ3_04665 was contiguous with two additional sequences with similar function. The first (SCAZ3_04660) contained an LPXTG-motif (a cell wall anchor domain). The second, according to the PGAAP annotation, was a common BLAST hit with the M protein from *S. pyogenes* (MGAS10270), and subsequent global nucleotide alignment showed 56.3% sequence identity between the sequences. However, the *S. canis* sequence contained a C insertion (site 746) that had shifted the reading frame. Although the insertion had disrupted the gene sequence in this strain, it does not preclude the presence of functional copies in other strains of *S. canis.* Together, these last three genes may play an important role in cell adherence possibly producing enhanced virulence of *S. canis* strains containing the plasmid.

Recently, Richards *et al*. [52] detected multiple copies of this plasmid (exact repeats) in a second strain of *S. agalactiae*: the bovine strain FSL S3-026. Designated FSL S3-026-S20, this copy of the plasmid showed 60.9% sequence identity (global alignment) with *S. canis.* There is strong differentiation between human and bovine *S. agalactiae* populations [52] and the *S. canis* strain studied here was isolated from bovine milk. Consequently, it seems plausible that the plasmid was exchanged between these species in the bovine environment. Indeed, out of the ten *S. agalactiae* genome sequences available, nine are human isolates and eight lack the plasmid. The ninth (NEM316), however, shows very high sequence identity for the plasmid when compared to *S. canis* (92.4%, global alignment), suggesting, on first consideration, that the plasmid may have been exchanged recently in the human environment. However, although NEM316 is usually listed as a human sourced isolate, Sørensen *et al*. [53] determined its origin to be unknown, and the observations that it can ferment lactose (human sourced isolates typically cannot) [53], and compared to other human sourced isolates, it grows well in milk (PD Pavinski Bitar unpublished data), suggest that the strain may have had a close association with the bovine environment. In addition, the strain is MLST sequence type 23, which occurs in both bovine and human environments [53-55].

### Phages

*S. canis* contained a 59 CDS prophage (Prophage 1, Figure 1) (see also Additional file 2: locus tags SCAZ3_03020 through SCAZ3_03310 [53,556 bp]). In general, the prophage had the distinctive modular arrangement of tailed phage structural genes described for lactic acid bacteria [56]. Putative *att* sites (a 12 bp direct repeat) were identified 776 bp upstream of SCAZ3_03020 (hypothetical cytosolic protein) and 133 bp downstream of SCAZ3_03310 (site-specific recombinase). Upstream of the site-specific recombinase were two genes characteristic of the lysis module (holin and lysin) and upstream of this were genes characteristic of the tail modules. Consequently, this end of the prophage likely contained the *attR* site. However, site-specific recombinase (present as two contiguous copies) belongs to the resolvase family of enzymes, and these enzymes usually occur in the lysogeny module [57], which typically occurs at the other end of the phage. In addition to phage structural genes, the prophage also contained five CDS that were homologous with virulence factors in the VFDB. SCAZ3_03175 (DNA-cytosine methyltransferase) was homologous with the same DNA methylase from *E. coli* as the methyltransferase gene within the integrative plasmid and therefore may provide the phage with similar protection from host restriction nucleases. Similarly, both the phage (SCAZ3_03220: ATP-dependent *clp* proteolytic subunit) and plasmid contained CDS that were homologous with *clp* genes from *L. monocytogenes*, which play a role in competence, development, and stress survival in *S. pneumoniae*[46]. SCAZ3_03045 (serine/threonine rich platelet-type antigen) was homologous with C protein alpha antigen (*bca*) from *S. agalactiae* (A909), which is important in the initial stages of mice infection [58]. Gene ontology (GO) terms for this CDS also suggest virulence, indicating that the gene product is a cell surface component that binds to calcium ions, and this molecular function can be linked to pathogenesis. The remaining two CDS homologous with virulence factors (SCAZ3_03050 and SCAZ3_03060) were insertion sequences (transposases) homologous to the *E. coli* virulence plasmid pB171. These findings indicate several similarities between phage and the integrative plasmid genes; possibly reflecting shared infection and survival characteristics between these two types of mobile genetic element.

Using BLASTn we detected the presence of the prophage in three additional *Streptococcus* species: *S. agalactiae* (strains S3-026 [bovine isolate] and A909 [human isolate]), *S. urinalis*, and *Streptococcus porcinus*. Subsequent global nucleotide alignment revealed high sequence identity with *S. agalactiae* (S3-026) (97.3%) and particularly with *S. urinalis* (99.4%) suggesting very recent exchange between *S. canis* and *S. urinalis*. Sequence identities for *S. agalactiae* (A909) and *S. porcinus* were 63.1% and 64.1% respectively, suggesting older exchanges. To the knowledge of the authors, *S. urinalis* has only been reported as being isolated from humans [59,60]. *S. canis* however, is typically found in animal hosts such as dogs and cats, but there are reports of human infection, usually ulcer or wound infection in patients who own domestic dogs [14-16]. Therefore, it’s possible that *S. canis* and *S. urinalis* exchanged the phage within a shared human environment. However, it’s also possible, that since *S. urinalis* is rare in humans, that a different, as yet unknown niche, is its principal habitat and that *S. canis* may be present in that same niche.

We also found evidence for a second prophage (~63 CDS) (Prophage 2, Figure 1). Although putative *attL/R* sites could not be found, the putative *attL* end was a site-specific recombinase (SCAZ3_03510), typical of the lysogeny module. BLASTn detected the phage in three additional *Streptococcus* species: *S. dysgalactiae* subsp. *equisimilis*, *S. pyogenes*, and *S. dysgalactiae* subsp. *dysgalactiae*. However, global nucleotide alignment revealed only moderate sequence identity to *S. canis*: 65.7%, 62.9%, and 58.0% respectively. Being the last of a generally contiguous sequence of phage genes for *S. canis*, *S. pyogenes*, and *S. dysgalactiae* subsp. *equisimilis*, and typical of the lysis module, a phage holin gene (SCAZ3_03820) was assumed to represent the *attR* end of the phage.

### Integrative conjugative element

*S. canis* also contained a contiguous section of 54 CDS (SCAZ3_05800 - SCAZ3_06105) (62,915 bp) (see Additional file 2) that was characteristic of an ICE. The section contained an integrase, three CDS homologous to the conjugative transposon Tn5252 (one of which was relaxase), Type IV secretory pathway genes belonging to the VirB4 family (implicated in conjugation) [61], and was flanked by putative *attL/R* sites (a 41 bp imperfect direct repeat that differed by 2 bp). However, unlike the ICE reported for numerous other *Streptococcus* species [62], the 5’ end was not inserted at the 3’ end of a tRNA or ribosomal gene, rather its 3’ end was inserted at the 5’ end of a ribosomal gene (ribosomal biogenesis GTPase). The ICE also possessed numerous additional genes characteristic of a mobile genetic element; for example, excisionase, helicase, abortive infection (Abi) system genes, and a zeta toxin gene characteristic of toxin-anti toxin (TA) systems, as well as a group II intron reverse transcriptase/maturase (SCAZ3_05875). In addition, the ICE contained three CDS that were homologous with virulence factors. Two of these CDS (agglutinin receptors, SCAZ3_05915 and SCAZ3_05930) were homologous with aggregation substance (AS) genes from *Enterococcus faecalis* plasmids. These genes (i) facilitate conjugative exchange by mediating cell binding, (ii) contribute to pathogenicity by enhancing cell adhesion and internalization, and (iii) favor intracellular survival within macrophages [63]. The third CDS (methyl transferase, SCAZ3_05815) was homologous with the same DNA methylase of *E. coli*, as for both the plasmid and phage, and therefore may provide the ICE with similar protection from host restriction nucleases.

A BLASTn search detected the ICE in two additional *Streptococcus* species: *S. agalactiae* (strains S3-026 and NEM316) and *S. dysgalactiae* subsp. *dysgalactiae.* Global nucleotide alignment showed these ICE to have only moderate identity with the *S. canis* ICE: 58.2%, 55.0%, and 60.1% respectively. In addition to the genes described, the *S. canis* ICE also contained the lactose operon Lac.2 [52,64], suggesting that the ability to ferment lactose may have been acquired via lateral gene transfer. Furthermore, Lac.2 is also contained within the *S. agalactiae* (NEM316) and *S. dysgalactiae* subsp. *dysgalactiae* ICE, suggesting that these strains may have also acquired the ability to ferment lactose via lateral gene transfer.

Given *S. canis* strain FSL S3-227’s association with the bovine environment, it is notable that there is a putative nisin resistance CDS (SCAZ3_06155) within the genome. Nisin is a lantibiotic produced by some strains of the mastitis causing pathogen *Streptococcus uberis*, and has been shown to provide these strains with a competitive advantage during intramammary infection when compared to non-producer strains [65]. The gene operon required for nisin production is also present in bovine isolates of *S. agalactiae*[52]. Although *S. canis* strain FSL S3-227 lacked this operon, the presence of a nisin resistance CDS might assist *S. canis* during intramammary infection.

### Population genetics

To assess the population genetic structure of *S. canis* we ribotyped an additional 82 isolates obtained from bovine, canine, and feline hosts (see Methods). Of these, a subset of 46 isolates was selected for multi locus sequence typing (see Methods). The ribotyping revealed a total of 17 ribotypes for all 83 isolates (Table 1). With one exception, isolates from multiple cows within each dairy herd belonged to a single ribotype per herd. This supports previous observations, which found that mastitis outbreaks due to *S. canis* were generally caused by a single strain within a herd [10,12], suggesting contagious transmission, exposure to a point-source, or host-adaptation of specific *S. canis* strains [66]. Among the 46 isolates selected for the MLST scheme, we identified 16 sequence types (STs) (see Additional file 5 for allelic profiles). Diversity among canine isolates was substantially higher than among bovine isolates (Table 2). For example, there were 14 canine STs (diversity: 0.90) compared to 3 bovine STs (diversity: 0.49). For the ribotypes, there were 13 canine ribotypes (diversity: 0.88) compared to 4 bovine ribotypes (diversity: 0.67). Nucleotide diversity showed a different pattern. Although values were similar, bovine diversity was slightly higher (canine: 0.0094, bovine: 0.0127). The distinctiveness of the canine and bovine isolates was confirmed by the Fisher exact test, which showed the frequency distribution of ribotypes and STs between canine and bovine isolates to be significantly different (*P* = 0.0000 [ribotypes], *P* = 0.0030 [STs]). An analysis of molecular variance (AMOVA) [67], however, did not detect any significant differentiation between these isolates (Φ_ST_ = 0.082, *P* = 0.052). 

**Table 1:** Isolate screening data

**ID**	**Host/Tissue**	**Herd/State/Country**	**N1**	**N2**	**Ribotype**	**ST**	**CC**
FSL Z3-022	Bovine	Not available, Belgium	1		116-1000-4	1	3
IT-SCA-35	Bovine	I-1, Italy	3		116-679-1	1	3
IT-SCA-65	Bovine	I-2, Italy	3		116-679-1	1	3
IT-SCA-73	Bovine	I-3, Italy	1		116-679-1	1	3
IT-SCA-31	Bovine	I-4, Italy	1		116-1000-4	1	3
IT-SCA-92	Bovine	I-5, Italy	3		116-1000-4	1	3
IT-SCA-80	Bovine	I-6, Italy	2		116-1000-4	2	3
IT-SCA-24	Bovine	I-7, Italy	3		116-679-1	1	3
FSL Z3-012*	Bovine	U-1, NY, USA	4		116-679-1	1	3
FSL Z3-006*	Bovine	U-2, NY, USA	4		116-679-1	1	3
R2-766	Bovine	U-3, NY, USA	5		116-679-1	1	3
FSL Z3-227*	Bovine	U-4, NY, USA	11		116-679-1	1	3
FSL Z3-316	Bovine	U-5, NY, USA	3	2^1^	116-1180-4	14	
FSL Z3-010	Bovine	U-6, NY, USA	4		116-975-3	14	
FSL Z3-346	Bovine	U-7, NY, USA	2		116-679-1	1	3
FSL Z3-013*	Bovine	U-8, NY, USA	2		116-975-3	14	
FSL Z3-015*	Bovine	U-9, NY, USA	1	1^2^	116-975-3	14	
FSL Z3-011*	Bovine	U-10, NY, USA	1		116-679-1	1	3
FSL Z3-234*	Bovine	U-10, NY, USA	2		116-975-3	14	
FSL Z3-023*	Canine, wound exudate	Not available, Belgium			1000-5	11	2
FSL Z3-046*	Canine, lip	NY, USA			1168-1	9	
FSL Z3-048	Canine, ear swab	NY, USA			1000-5	11	2
FSL Z3-049	Canine, ear swab	NY, USA			1000-4	1	3
FSL Z3-050	Canine, vaginal swab	NY, USA			1000-4	1	3
FSL Z3-053	Canine, vaginal swab	NY, USA			679-1	1	3
FSL Z3-054	Canine, vaginal swab	NY, USA			679-1	1	3
FSL Z3-057	Canine, hock abscess	NY, USA			975-3	15	
FSL Z3-058	Canine, ear swab	NY, USA			1171-2	6	
FSL Z3-116	Canine, pharyngeal swab	NY, USA			1168-5	8	
FSL Z3-117	Canine, ear swab	NY, USA			1000-5	12	2
FSL Z3-118	Canine, ear swab	NY, USA			697-1	1	3
FSL Z3-119	Canine, ear swab	NY, USA			1000-4	9	4
FSL Z3-120	Canine, vaginal swab	NY, USA			679-1	1	3
FSL Z3-121*	Canine, vaginal swab	NY, USA			1173-7	4	1
FSL Z3-154*	Canine, vaginal swab	NY, USA			1173-8	10	4
FSL Z3-155	Canine, urine	NY, USA			679-1	1	3
FSL Z3-156	Canine, throat	NY, USA			1168-1	10	4
FSL Z3-157*	Canine, pharyngeal swab	NY, USA			1174-4	16	1
FSL Z3-158	Canine, pharyngeal swab	NY, USA			1174-4	5	1
FSL Z3-159	Canine, eye	NY, USA			679-1	3	3
FSL Z3-160	Canine, vaginal swab	NY, USA			679-1	3	3
FSL Z3-162*	Canine, ear swab	NY, USA			1174-7	13	2
FSL Z3-163	Canine, dermis	NY, USA			679-1	1	3
FSL Z3-165	Canine, vaginal swab	NY, USA			1000-5	11	2
FSL Z3-166*	Canine, pharyngeal swab	NY, USA			1170-7	7	
FSL Z3-007*	Feline	U-4, NY, USA			679-1	1	3

N1 = Number of isolates from each herd ribotyped (either individual cow or quarter milk sample). For each herd, only one isolate representing a distinct ribotype was typed using MLST.

N2 = Number of isolates from milking machine rubber liners or bulk tank milk.

ST = sequence type.

CC = clonal complex.

^1^ Isolated from milking machine rubber liners.

^2^ Isolated from bulk tank milk.

* Isolate contains plasmid (see text).

**Table 2:** Isolate diversity indices and summary statistics

	***n*-RT**	**RT**	**RT-*h***	***n*-ST**	**ST**	**ST-*h***	**θ**	**π**	**plasmid**
All	83	17	0.90	46	16	0.76	0.0127	0.0111	15
Bovine*	56	4	0.67	19	3	0.49	0.0089	0.0127	7
Canine	26	13	0.88	26	14	0.90	0.0139	0.0094	7
Feline	1	1		1	1				1

*n*-RT = number of isolates ribotyped.

*n*-ST = number of isolates sequence typed.

RT = number of ribotypes.

RT-*h* = ribotype (gene) diversity.

ST = number of STs.

ST-*h* = ST (gene) diversity.

θ = population parameter theta (per site).

π = nucleotide diversity.

plasmid = number of strains containing the plasmid.

*The bovine isolates represent 18 distinct herds (farms). With one exception a single ST was obtained from each herd (two STs were obtained from one herd) (see Methods).

**Figure 3:**
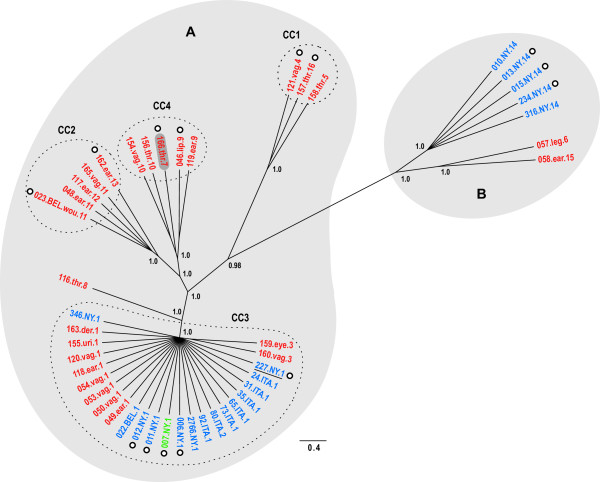
**ClonalFrame 75% majority-rule consensus phylogeny (node posterior probabilities are at least 0.75).** Posterior probabilities for major lineages are shown at nodes. Dashed circles show each clonal complex (CC) and grey shading shows isolates assigned to the two clusters (**A** and **B**) determined by the Structure analysis. Taxa labels are colored as follows: red = canine isolate, blue = bovine isolate, green *=* feline isolate. The first number in the label shows isolate ID. For canine isolates, tissue source follows the isolate ID, which is followed by the ST. Tissue source abbreviations are as follows: thr = throat, vag = vaginal, uri = urine, der = dermis, wou = wound exudate. For bovine and feline isolates, the ID is followed by the geographic location of collection (ITA = Italy, BEL = Belgium, NY = New York state, USA). Strain 227.NY.1 (underlined) is the strain who’s genome was sequenced in this study. Circles with white centers indicate those strains that contained the plasmid discussed in the text. The strain shaded in dark grey (166.thr.7) was grouped with CC4 members based on ClonalFrame analysis but it was not contained within CC4 based on eBURST.

**Figure 4:**
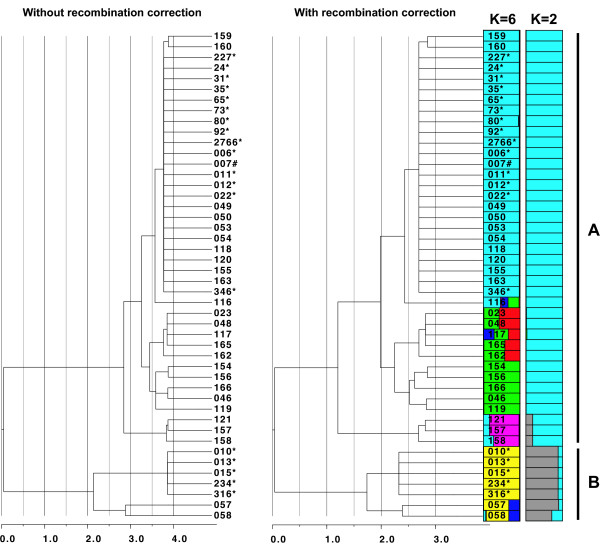
**ClonalFrame 75% majority-rule consensus phylogenies with and without correction for recombination.** The scale shows time in coalescent units. The phylogeny with recombination correction also shows for each isolate its proportion of ancestry for each genetic cluster determined by the Structure analyses. For *K* = 2 and *K* = 6, the different colors represent each cluster. The proportion of color shading for each bar represents the proportion of ancestry for the respective cluster. Vertical bars show the isolates assigned to clusters **A** and **B** when *K* = 2. Asterisk refers to bovine isolates; # refers to feline isolate.

Examination of evolutionary relationships among STs using a Bayesian phylogenetic approach (ClonalFrame, [68]) produced a well-supported phylogeny (Figure 3), with three independent runs of the Markov chain all producing congruent topologies. Repeating the runs without the recombination model (we assume no recombination) had no affect on the topology, but branch lengths did vary (Figure 4). The average total branch length for the three phylogenies, not accounting for recombination (15.9 coalescent time units), was slightly larger than the average length of the three phylogenies that did account for recombination (14.2 coalescent time units). 

The amount of recombination in bacteria can be quantified using two ratios: (i) the ratio of the frequency at which recombination occurs relative to mutation (*ρ*/*θ*), and (ii) the ratio of the rates at which nucleotides become substituted as a result of recombination and mutation (*r*/*m*). The latter ratio accounts for length and nucleotide diversity of imported fragments and therefore contains more information regarding the evolutionary impact of recombination [69]. Using ClonalFrame, we calculated these ratios to be: *ρ*/*θ* = 0.1 and *r*/*m* = 1.5, with the latter ratio indicating that recombination exceeded point mutation. Vos and Didelot [70] calculated *r*/*m* for 48 diverse species of bacteria, and their results revealed a wide range of values (63.6 – 0.02). *r*/*m* for *S. canis* ranked 25^th^ in this distribution (approximately in the middle). However, the average of the 48 rates was 7.7, suggesting a below average rate of recombination for *S. canis* when compared to these species of bacteria. When compared to the two *Streptococcus* species in the distribution, *S. canis* was much lower: *S. pneumoniae* = 23.1 (6^th^), *S. pyogenes* = 17.2 (8^th^). Similar results were obtained when *ρ*/*θ* for *S. canis* was compared to other *Streptococcus* species: *S. uberis* = 17.2 [71], *S. pneumoniae* = 23.1 [72].

We expanded the evolutionary analysis by also applying the parsimony-based approach e-BURST [73], which explores fine scale evolutionary relationships among STs. The ClonalFrame phylogeny and e-BURST results were generally concordant regarding the grouping of STs (Figure 3). The only discrepancy was ST7, which showed an intermediate relationship between STs 9 and 10 in the phylogeny, but was not grouped within the same clonal complex (CC) as STs 9 and 10 (ST7 was not grouped into any of the four clonal complexes).

Population structure was further examined using the Bayesian clustering approach implemented in the program Structure [74,75]. The number of clusters *K* was estimated by calculating the *ad hoc* statistic Δ*K*, which is a measure of the second order rate of change of the probability of the data *L*(*K*) for each value of *K*[76] (see Methods for a full explanation of the approach). The analysis showed the optimum number of genetic clusters (*K*) to be two (A and B) (Figure 3 and Additional file 6). All four clonal complexes and ST8 were grouped into cluster A, whereas cluster B contained STs 6, 14, and 15. The ClonalFrame phylogeny showed CC1 to be the most closely related lineage to cluster B; concordantly, this lineage shared a small proportion (~12%) of its ancestry with cluster B (Figure 4). Although Δ*K* indicated that *K* was two and the Ln *P*(*D*) scores plateaued for *K* values of two, three, and four (see Additional file 6), the Ln *P*(*D*) scores rose slightly after *K* = 4 and again plateaued starting with *K* = 6. This suggests a pattern of hierarchal differentiation among isolates, with further subdivision present within clusters. Assuming *K* = 6 for this additional subdivision, the assignment of individuals (proportion of ancestry) into these clusters delineated isolates into groups concordant with the six major lineages seen in the ClonalFrame phylogeny (Figure 4).

Only three (1, 2, and 14) of the 16 STs were found in bovines, and one of these (ST2) was a single locus variant of the predominant ST in cattle (ST1). Consequently, there was a much higher diversity of STs found in canine, producing a significant differentiation in the frequency of STs between the two hosts. Previous studies have shown the incidence of *S. canis* isolation from bovine to be rare [77-82]. This observation coupled with the relatively low diversity of bovine STs suggests a recent adaptation to the bovine environment. Thus, the MLST data, the genomic features shared between *S. canis* and other bovine adapted *Streptococcus* species discussed earlier, and the epidemiological information associated with the original study regarding this strain [12], suggest that ST1 could be bovine adapted. The AMOVA, however, did not detect any significant differentiation between hosts. This is likely due to the fact that this analysis incorporates genetic distance and the strongest signal of differentiation (as detected by the Structure analysis) was between clusters A and B (Figure 3), both of which contain a bovine-associated ST (ST1 and ST14, respectively). This result does not necessarily preclude a very recent adaptation to the bovine environment for specific STs/lineages. If the adaptation were very recent, any phylogenetic signal recovered from the ST sequence data resulting from host partitioning would be very weak. Examination of the phylogeny (Figure 3) shows STs 1 and 2 to be closely related and contained within CC3, whereas ST14 is one of the most divergent ST from CC3. Given the above reasoning, this observation suggests that recent adaptation to the bovine environment must have occurred independently in these two lineages. A similar scenario was recently proposed for *S. agalactiae* where virulent lineages independently evolved from an ancestral core that were specific to human or bovine hosts [53].

There is, however, a possible alternative interpretation, that is contrary to the recent bovine adaptation argument. The most frequent ST was clearly ST1 (*n =* 22, 48% of isolates). This ST was also seen in all three hosts (canine, bovine, feline), including a wide range of canine tissue types (vaginal, ear, skin, urine, eye), and also all three sampling locations (USA, Italy, and Belgium). In contrast, with one exception, no other ST was seen in more than one host or geographic location. The exception was ST11, which was seen in both USA and Belgium. These observations suggest that ST1 is the most ancestral ST in the data set [83,84], and also possibly a generalist, with the ability to infect different hosts and tissue types. Genomic comparisons showed that strain FSL S3-227 shared multiple mobile genetic elements with *S. agalactiae* and *S. dysgalactiae* subsp. *dysgalactiae* strains isolated from the bovine environment, with one of these elements (the ICE) showing high sequence divergence. Although the ICE contained the Lac.2 operon, suggesting that this LGT may have contributed to bovine adaptation, the high divergence and multiple additional LGTs suggest that *S. canis* ST1 may have had an extended association with the bovine environment, arguing against more recent adaptation. Consequently, if ST1’s lineage has possessed the ability to infect cows for an extended period of time, and is also the most ancestral with all lineages having descended from it, in order for the ST14 lineage to have recently acquired the ability to infect cows, all lineages intermediate between ST1 and ST14 must have previously lost this ability. This might have occurred as a single event on the branch connecting CC3 to ST8. Alternatively, all strains are generalist and the more recent lineages have simply had insufficient time to encounter the bovine environment and/or that our sample size was too low to detect their presence.

The distribution of the plasmid provides yet another perspective. The plasmid has only been observed in one additional species: *S. agalactiae* (strain FSL-S3026 [isolated from a bovine host], and strain NEM316 [potential association with the bovine environment]). Therefore, it is possible that the plasmid was exchanged between *S. canis* and *S. agalactiae* in the bovine environment, however, the plasmid appears randomly distributed among *S. canis* isolates, regardless of host species or ST. For example, (i) a Fisher exact test showed no significant difference in its distribution between bovine and canine isolates (*P* = 1.0), (ii) it was present in all clonal complexes and clusters, and (iii) it was present in all three hosts including a wide range of canine tissue types (vaginal, ear, throat, lip). Consequently, the plasmid appears to have moved freely between bovine and canine environments, supporting the generalist argument. An alternative explanation is that *S. canis* may have obtained the plasmid on independent occasions from one or more different hosts. A similar process involving various mobile genetic elements has been observed for various *Streptococcus* species [17,85,86].

## Conclusion

Characterization of the genome sequence for *S. canis* strain FSL S3-227 detected a high diversity of virulence factors. Approximately three quarters of the genes that were homologous to known *Streptococcus* virulence factors are involved in invasion, evasion, and colonization, perhaps explaining *S. canis*'s ability to infect a wide range of tissue types. Furthermore, the putative ancestral clonal complex (accounting for more than half of collected isolates) occurred in a wide range of tissue types, all hosts, and all geographic locations suggesting a wide and diverse niche. It has been demonstrated that the source of bovine *S. canis* infection can be other farm-yard animals such as domestic cats [12]. Our results, revealed high genetic similarity among bovine, feline, and canine sourced isolates further supporting domestic farm-yard animals as infection sources. Despite the modest level of recombination for *S. canis* when compared to other *Streptococcus* species, LGT is still clearly an important evolutionary phenomenon in this species as evidenced by the multiple MGE present within its genome (i.e. plasmid, phage, and ICE) and the occurrence of an integrative plasmid in approximately half of the collected isolates. Furthermore, the evidence for LGT between *S. canis* and two additional bovine mastitis causing pathogens (*S. agalactiae*, and *S. dysgalactiae* subsp. *dysgalactiae*) suggests a close association with the bovine environment for *S. canis*, with this LGT possibly contributing to adaptation to this environment. Many virulence factors are also carried within these MGE, further highlighting the importance of these mobile elements in the evolution of this pathogen. Furthermore, the high frequency of virulence factors within multiple MGE, coupled with LGT between *S. canis* and a human sourced bacteria (*S. urinalis*), suggests the possibility for additional transport of virulence factors into the human environment.

## Methods

### Strain selection, sequencing, and assembly

*S. canis* strain FSL Z3-227 was isolated from a composite milk sample obtained from a cow with an intra-mammary infection. The sample was collected on the 6^th^ of April 1999 from a cow located in central New York State within a dairy herd experiencing an outbreak of *S. canis* induced mastitis. Bacterial culture and ribotyping results indicated that a farm cat with chronic sinusitus was the likely source of the outbreak [12]. Utilizing a seven-gene MLST scheme developed here (see below), strain FSL Z3-227 was determined to be ST1. This ST was associated with multiple host species (bovine, canine, feline). In addition, it was the most common ST among bovine isolates and the only ST to be found in all three countries represented in the study. Therefore, it was thought to have the potential to have a broad complement of virulence factors, including those potentially associated with niche adaptation in cattle, and was consequently selected for genome sequencing.

Roche/454 pyrosequencing was used to determine the genome sequence, and Newbler v1.1 (454 Life Sciences Corporation) was used to assemble the reads. Using restriction enzyme BgIII, an optical map of the genome was built by OpGen Technologies, Inc. (Madison, WI). Scaffold order and orientation was determined by alignment to the optical map using Opgen Mapviewer. Small inter and intra-scaffold gaps were closed by PCR and Sanger sequencing. Seven larger gaps were closed using long range PCR and Illumina sequencing. Illumina reads were assembled using Velvet [87], and the optimum assembly was determined using the N50 statistic. Annotation of the genome assembly was performed using the NCBI Prokaryotic Genomes Automatic Annotation Pipeline (PGAAP) and Blast2GO v.2.5.0 (*E* value cut-off = 1e-6 and minimum amino acid alignment length cut-off [hsp-length] = 33) [88] (annotations are shown in Additional file 2). This Whole Genome Shotgun project has been deposited at DDBJ/EMBL/GenBank under the accession AIDX00000000. The version described in this paper is the first version, AIDX01000000.

### Homologous gene clustering

The MCL algorithm [89] as implemented in the MCLBLASTLINE pipeline (available at http://micans.org/mcl) was used to delineate homologous protein sequences among 214 *Streptococcus* genomes including *S. canis* (see Additional file 3). Based on sequence similarity, the pipeline uses Markov clustering (MCL) to assign genes to homologous clusters. Similarity was obtained from a reciprocal BLASTp within and between all genome pairs using an *E* value cut-off of 1e-5. The MCL algorithm was implemented using an inflation parameter of 1.8. Simulations have shown this value to be generally robust to false positives and negatives [90].

### Virulence factors

Amino acid sequences for all *S. canis* CDS were searched against the VFDB using BLASTp. We used an *E* value cut-off of 1e-5 and retained the single best hit. The search was refined by repeating the BLASTp search against a database that contained only *Streptococcus* virulence factors (88 genes).

### Population genetics

Including the strain genome sequenced here, a total of 83 *S. canis* isolates were obtained from bovine (*n =* 56), canine (*n =* 26), and feline (*n =* 1) hosts (Table 1). Isolates of canine/feline origin included 25 canine isolates from patients of Cornell University’s College of Veterinary Medicine, Ithaca, NY, USA, one canine isolate from Belgium, and one isolate from a cat living on a dairy farm in upstate New York. The feline isolate was the likely source of a mastitis outbreak at the same farm. Canine isolates from NY originated from dermis (*n =* 1), ear swabs (*n =* 7), eye (*n =* 1), hock abscess (*n =* 1), lip (*n =* 1), pharyngeal swabs (*n =* 5), urine (*n =* 1), and vaginal swabs (*n =* 8), and were collected from December 2003 to May 2004. The canine isolate from Belgium originated from wound exudate [1] and the feline isolate originated from a nasal swab taken from a cat with chronic sinusitis [12]. Bovine isolates originated from one herd in Belgium (from mastitic milk; [1], seven dairy herds in Italy (16 isolates from bovine milk; collected in 2003 and 2004), and 10 dairy herds in NY (two isolates from milking machine liners, one isolate from bulk tank milk, and 34 cow milk isolates; collected from 1999 to 2005). In addition to strain FSL Z3-227, all 82 isolates were ribotyped using the commercial RiboPrinter system with EcoRI.

Single isolates representing the ribotypes seen in each herd (two isolates from the herd U-10 and a single isolate from each of the remaining herds) (*n =* 19) were combined with all canine/feline isolates (*n =* 27) and further screened using a seven housekeeping MLST scheme with PCR primers previously used for characterization of *S. pyogenes, S. pneumoniae,* or *S. uberis*[91-95]. See Additional file 7 for primer sequences and PCR profiles. MLST allele sequences were aligned using MAFFT v6.814b [96] as implemented in Geneious v5.1.2. Isolate genetic diversity indices were calculated using the program DNASP version 4.0 [97]. Diversity indices among STs were obtained by concatenating the seven alleles (4,014 bp). Diversity among ribotypes and STs was calculated using the formula for haplotype (gene) diversity [97]. Again using the concatenated allele sequences, population differentiation between bovine and canine groupings of isolates (bovine = 19 canine = 26) was determined by assessing the frequency distribution of STs (Fisher exact test) between the groups. Differentiation was also determined by an AMOVA as implemented in Arlequin v3.11 [98]. The AMOVA differs from the exact test because in addition to assessing ST frequency distribution, it also considers genetic divergence among isolate sequences in its determination of differentiation.

With the exception of strain FSL Z3-227 (our genome sequence), all isolates typed using the MLST scheme (*n =* 45) were also PCR screened for the presence of a 55 CDS plasmid (see Results and discussion). Presence/absence of the plasmid was determined using 25 primer pairs that were contiguous along the length of the plasmid (see Additional file 8).

Evolutionary relationships among STs were examined using eBURSTv3 [73]. STs were grouped into clonal complexes and support for complex founders was estimated using 1000 bootstrap replicates. We used the most stringent (default) eBURST setting for grouping STs into a complex, where STs within the same complex shared identical alleles at ≥ six of the seven loci with at least one other member of the complex.

Deeper evolutionary relationships (among clonal complexes for example) were inferred using the Bayesian phylogenetic approach implemented in ClonalFrame v1.1 [68]. This approach incorporates a model that attempts to account for recombination. The Markov chain was run with 1,000,000 iterations after an initial burn-in of 50,000 iterations. Three independent runs were used to assess topological convergence. To assess the effect of recombination, all runs were repeated with the recombination rate parameter (R) held at zero (i.e. the effect of recombination on the topology was not accounted for). We used ClonalFrame to calculate the recombination ratios *ρ*/*θ* and *r*/*m* (average of the three runs).

Isolates were clustered using the Bayesian approach implemented in Structure v2.3.2 [74]. The number of clusters *K* was estimated by calculating the *ad hoc* statistic Δ*K*[76]. Δ*K* was calculated for *K* = 1 through 10 using 5 Markov chains for each value of *K*. The simulations of Evanno *et al*. [76] showed that the highest value for Δ*K* reliably identified the optimum value of *K*. Chains were run for 500,000 steps following an initial burn-in of 100,000 steps, using the admixture ancestry and correlated allele frequency models. Once the optimum value of *K* was identified, strains were assigned to clusters using assignment coefficients (proportion of cluster membership) generated from an additional run utilizing the linkage ancestry and correlated allele frequency models. A study of recombinant bacterial populations showed the linkage model of ancestry to produce the most accurate assignment scores in situations where there are multiple linked loci along contiguous sections of DNA [75]. The model assumes these sections, which could be recombinant, to be discrete units of inheritance. Markov chains were run for 2,000,000 steps following an initial burn-in of 500,000 steps.

## Competing interests

The authors declare that they have no competing interests.

## Authors’ contributions

VPR conducted data analysis and wrote the manuscript; MJS provided the conceptual framework, experimental design, and helped write the manuscript; PDPB and PL conducted laboratory work associated with genome sequencing; TL conducted data analysis and genome assembly; BW conducted laboratory work associated with the survey of plasmid distribution across canine and bovine isolates; LT, and PM conducted field work associated with population genetics; RNZ conceived of the field and laboratory work for population genetics, conducted MLST and ribotyping, and was involved in manuscript preparation. All authors read and approved the final manuscript.

## Supplementary Material

Additional file 1 ***Streptococcus *RefSeq genome summary statistics.** Click here for file

Additional file 2 ***S. canis *annotation.** Click here for file

Additional file 3 **Additional *Streptococcus *genomes.** Click here for file

Additional file 4 Insertion sites of putative integrative plasmid. Click here for file

Additional file 5 ***S. canis *isolate MLST allele data.** Click here for file

Additional file 6 **Ln *P(D) *scores for Structure analysis.** Click here for file

Additional file 7 MLST PCR primer details. Click here for file

Additional file 8 Putative integrative plasmid PCR primer details. Click here for file
