# Supplementary material for: Genome characterization and population genetic structure of the zoonotic pathogen, Streptococcus canis
Source: BMC Microbiol. 2012 Dec 18;12:293. doi: 10.1186/1471-2180-12-293 (PMC3541175; doi:10.1186/1471-2180-12-293)
Supplement: Additional file 1 — Streptococcus RefSeq genome summary statistics. [file 1471-2180-12-293-S1.doc]

**Additional file 1.** Streptococcus RefSeq genome summary statistics

| ACCESSION | ORGANISM | bp | CDS | tRNA | rRNAoperon | %GC |
| --- | --- | --- | --- | --- | --- | --- |
| AEXT01000000 | *S. agalactiae* FSL S3-026 | 2455848 | 2319 | 70 | 6(7) | 36.1 |
| NC_009009 | *S. sanguinis* SK36 | 2388435 | 2270 | 61 | 4 | 43.4 |
| NC_015215 | *S. gallolyticus* ATCC BAA 2069 uid63617 | 2356444 | 2309 | 80 | 7 | 37.6 |
| NC_013798 | *S. gallolyticus* UCN34 uid46061 | 2350911 | 2223 | 71 | 6 | 37.6 |
| AIDX02000000 | *S. canis* | 2267856 | 2212 | 67 | 6 | 39.7 |
| NC_012471 | *S. equi* subsp. *equi* 4047 | 2253793 | 2001 | 66 | 6 | 41.3 |
| NC_010380 | *S. pneumoniae* Hungary19A-6 | 2245615 | 2155 | 55 | 4 | 39.6 |
| NC_011900 | *S. pneumoniae* ATCC 700669 | 2221315 | 1990 | 58 | 4 | 39.5 |
| NC_015760 | *S. salivarius* JIM8780 uid70481 | 2217184 | 2027 | 68 | 6 | 39.9 |
| NC_004368 | *S. agalactiae* NEM316 | 2211485 | 2094 | 80 | 7 | 35.6 |
| NC_010582 | *S. pneumoniae* CGSP14 | 2209198 | 2206 | 58 | 4 | 39.5 |
| NC_009785 | *S. gordonii* str. Challis substr. CH1 | 2196662 | 2051 | 59 | 4 | 40.5 |
| NC_015875 | *S. pseudopneumoniae* IS7493 uid71153 | 2190731 | 2230 | 41 | 1 | 39.8 |
| NC_012468 | *S. pneumoniae* 70585 | 2184682 | 2202 | 58 | 4 | 39.7 |
| NC_003028 | *S. pneumoniae* TIGR4 | 2160842 | 2105 | 58 | 4 | 39.7 |
| NC_004116 | *S. agalactiae* 2603V R | 2160267 | 2124 | 80 | 7 | 35.6 |
| NC_015678 | *S. parasanguinis* ATCC 15912 uid49313 | 2153652 | 2022 | 61 | 4 | 41.7 |
| NC_012470 | *S. equi* subsp. *zooepidemicus* | 2149868 | 1869 | 57 | 5 | 41.5 |
| NC_013853 | *S. mitis* B6 | 2146611 | 2004 | 61 | 4 | 40 |
| NC_012926 | *S. suis* BM407 | 2146229 | 1932 | 52 | 4 | 41.1 |
| NC_015558 | *S. parauberis* KCTC 11537 uid67355 | 2143887 | 1868 | 54 | 5 | 35.5 |
| NC_007432 | *S. agalactiae* A909 | 2127839 | 1996 | 80 | 7 | 35.6 |
| NC_012466 | *S. pneumoniae* JJA | 2120234 | 2123 | 58 | 4 | 39.7 |
| NC_012469 | *S. pneumoniae* Taiwan19F-14 | 2112148 | 2044 | 58 | 4 | 39.8 |
| NC_012467 | *S. pneumoniae* P1031 | 2111882 | 2073 | 58 | 4 | 39.7 |
| NC_012891 | *S. dysgalactiae* subsp. *equisimilis* GGS 124 | 2106340 | 2095 | 57 | 5 | 39.6 |
| NC_015600 | *S. pasteurianus* ATCC 43144 uid68019 | 2100077 | 1869 | 60 | 5 | 37.4 |
| NC_009442 | *S. suis* 05ZYH33 | 2096309 | 2186 | 56 | 4 | 41.1 |
| NC_012924 | *S. suis* SC84 | 2095898 | 1898 | 56 | 4 | 41.1 |
| NC_009443 | *S. suis* 98HAH33 | 2095698 | 2185 | 56 | 4 | 41.1 |
| NC_011072 | *S. pneumoniae* G54 | 2078953 | 2115 | 58 | 4 | 39.6 |
| NC_008533 | *S. pneumoniae* D39 | 2046115 | 1914 | 58 | 4 | 39.7 |
| NC_003098 | *S. pneumoniae* R6 | 2038615 | 2042 | 58 | 4 | 39.7 |
| NC_004350 | *S. mutans* UA159 | 2030921 | 1960 | 65 | 5 | 36.8 |
| NC_011134 | *S. equi* subsp. *zooepidemicus* MGCS10565 | 2024171 | 1893 | 57 | 5 | 41.8 |
| NC_013928 | *S. mutans* NN2025 uid46353 | 2013587 | 1895 | 65 | 5 | 36.8 |
| NC_012925 | *S. suis* P1 7 | 2007491 | 1824 | 56 | 4 | 41.3 |
| NC_015291 | *S. oralis* Uo5 uid65449 | 1958690 | 1907 | 61 | 4 | 41.1 |
| NC_008024 | *S. pyogenes* MGAS10750 | 1937111 | 1979 | 63 | 6 | 38.3 |
| NC_008022 | *S. pyogenes* MGAS10270 | 1928252 | 1986 | 63 | 6 | 38.4 |
| NC_004070 | *S. pyogenes* MGAS315 | 1900521 | 1865 | 67 | 6 | 38.6 |
| NC_006086 | *S. pyogenes* MGAS10394 | 1899877 | 1886 | 67 | 6 | 38.7 |
| NC_007296 | *S. pyogenes* MGAS6180 | 1897573 | 1894 | 65 | 6 | 38.4 |
| NC_003485 | *S. pyogenes* MGAS8232 | 1895017 | 1839 | 67 | 6 | 38.5 |
| NC_004606 | *S. pyogenes* SSI-1 | 1894275 | 1859 | 57 | 5 | 38.6 |
| NC_008023 | *S. pyogenes* MGAS2096 | 1860355 | 1898 | 63 | 6 | 38.7 |
| NC_008532 | *S. thermophilus* LMD-9 | 1856368 | 1709 | 67 | 6 | 39.1 |
| NC_002737 | *S. pyogenes* M1 GAS | 1852441 | 1696 | 60 | 6 | 38.5 |
| NC_012004 | *S. uberis* 0140J | 1852352 | 1762 | 59 | 5 | 36.6 |
| NC_009332 | *S. pyogenes* str. Manfredo | 1841271 | 1745 | 66 | 6 | 38.6 |
| NC_007297 | *S. pyogenes* MGAS5005 | 1838554 | 1865 | 67 | 6 | 38.5 |
| NC_008021 | *S. pyogenes* MGAS9429 | 1836467 | 1877 | 67 | 6 | 38.5 |
| NC_011375 | *S. pyogenes* NZ131 | 1815785 | 1700 | 66 | 6 | 38.6 |
| NC_006448 | *S. thermophilus* LMG 18311 | 1796846 | 1889 | 67 | 6 | 39.1 |
| NC_006449 | *S. thermophilus* CNRZ1066 | 1796226 | 1915 | 67 | 6 | 39.1 |
