# Supplementary material for: Genome characterization and population genetic structure of the zoonotic pathogen, Streptococcus canis
Source: BMC Microbiol. 2012 Dec 18;12:293. doi: 10.1186/1471-2180-12-293 (PMC3541175; doi:10.1186/1471-2180-12-293)
Supplement: Additional file 4 — Insertion sites of putative integrative plasmid. [file 1471-2180-12-293-S4.doc]

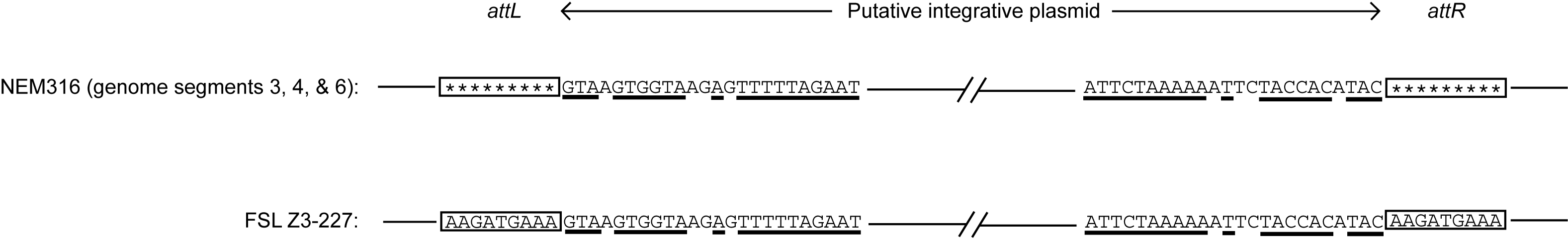


**Additional file 4.** Insertion sites of the putative integrative plasmid within *Streptococcus canis* (FSL Z3-227) and *Streptococcus agalactiae* (NEM316). The plasmid occurs in three separate genomic regions within NEM316 (segments 3, 4, and 6). Putative *attL* and *attR* sites are boxed. The *att* sites differed for each plasmid copy within NEM316 repeat and are shown as stars. The last 24bp at the terminal ends of each repeat were imperfect inverted repeats of themselves. Bold underlining shows the perfectly repeated nucleotides.
