# Supplementary material for: Genome characterization and population genetic structure of the zoonotic pathogen, Streptococcus canis
Source: BMC Microbiol. 2012 Dec 18;12:293. doi: 10.1186/1471-2180-12-293 (PMC3541175; doi:10.1186/1471-2180-12-293)
Supplement: Additional file 5 — S. canis isolate MLST allele data. [file 1471-2180-12-293-S5.doc]

**Additional file 5.** Isolate MLST allele data

| FSL-ID | ST | cpn60 | gapC | gki | gtr | mutS | recP | tuf |
| --- | --- | --- | --- | --- | --- | --- | --- | --- |
| Z3-022 | 1 | 1 | 1 | 1 | 3 | 1 | 1 | 1 |
| IT-SCA-35 | 1 | 1 | 1 | 1 | 3 | 1 | 1 | 1 |
| IT-SCA-65 | 1 | 1 | 1 | 1 | 3 | 1 | 1 | 1 |
| IT-SCA-73 | 1 | 1 | 1 | 1 | 3 | 1 | 1 | 1 |
| IT-SCA-31 | 1 | 1 | 1 | 1 | 3 | 1 | 1 | 1 |
| IT-SCA-92 | 1 | 1 | 1 | 1 | 3 | 1 | 1 | 1 |
| IT-SCA-80 | 2 | 1 | 1 | 1 | 3 | 1 | 1 | 6 |
| IT-SCA-24 | 1 | 1 | 1 | 1 | 3 | 1 | 1 | 1 |
| Z3-012 | 1 | 1 | 1 | 1 | 3 | 1 | 1 | 1 |
| Z3-006 | 1 | 1 | 1 | 1 | 3 | 1 | 1 | 1 |
| R2-766 | 1 | 1 | 1 | 1 | 3 | 1 | 1 | 1 |
| Z3-227 | 1 | 1 | 1 | 1 | 3 | 1 | 1 | 1 |
| Z3-316 | 14 | 8 | 4 | 6 | 6 | 6 | 6 | 5 |
| Z3-010 | 14 | 8 | 4 | 6 | 6 | 6 | 6 | 5 |
| Z3-346 | 1 | 1 | 1 | 1 | 3 | 1 | 1 | 1 |
| Z3-013 | 14 | 8 | 4 | 6 | 6 | 6 | 6 | 5 |
| Z3-015 | 14 | 8 | 4 | 6 | 6 | 6 | 6 | 5 |
| Z3-011 | 1 | 1 | 1 | 1 | 3 | 1 | 1 | 1 |
| Z3-234 | 14 | 8 | 4 | 6 | 6 | 6 | 6 | 5 |
| Z3-023 | 11 | 7 | 1 | 2 | 1 | 3 | 3 | 2 |
| Z3-046 | 9 | 6 | 1 | 4 | 1 | 3 | 4 | 4 |
| Z3-048 | 11 | 7 | 1 | 2 | 1 | 3 | 3 | 2 |
| Z3-049 | 1 | 1 | 1 | 1 | 3 | 1 | 1 | 1 |
| Z3-050 | 1 | 1 | 1 | 1 | 3 | 1 | 1 | 1 |
| Z3-053 | 1 | 1 | 1 | 1 | 3 | 1 | 1 | 1 |
| Z3-054 | 1 | 1 | 1 | 1 | 3 | 1 | 1 | 1 |
| Z3-057 | 15 | 9 | 5 | 6 | 7 | 9 | 7 | 5 |
| Z3-058 | 6 | 3 | 5 | 1 | 4 | 8 | 5 | 5 |
| Z3-116 | 8 | 5 | 2 | 1 | 1 | 7 | 2 | 1 |
| Z3-117 | 12 | 7 | 1 | 2 | 1 | 3 | 8 | 2 |
| Z3-118 | 1 | 1 | 1 | 1 | 3 | 1 | 1 | 1 |
| Z3-119 | 9 | 6 | 1 | 4 | 1 | 3 | 4 | 4 |
| Z3-120 | 1 | 1 | 1 | 1 | 3 | 1 | 1 | 1 |
| Z3-121 | 4 | 2 | 3 | 3 | 2 | 2 | 1 | 7 |
| Z3-154 | 10 | 6 | 1 | 4 | 1 | 5 | 4 | 4 |
| Z3-155 | 1 | 1 | 1 | 1 | 3 | 1 | 1 | 1 |
| Z3-156 | 10 | 6 | 1 | 4 | 1 | 5 | 4 | 4 |
| Z3-157 | 16 | 10 | 3 | 3 | 2 | 2 | 1 | 7 |
| Z3-158 | 5 | 2 | 3 | 1 | 2 | 2 | 1 | 7 |
| Z3-159 | 3 | 1 | 1 | 1 | 5 | 1 | 1 | 1 |
| Z3-160 | 3 | 1 | 1 | 1 | 5 | 1 | 1 | 1 |
| Z3-162 | 13 | 7 | 1 | 2 | 1 | 3 | 4 | 2 |
| Z3-163 | 1 | 1 | 1 | 1 | 3 | 1 | 1 | 1 |
| Z3-165 | 11 | 7 | 1 | 2 | 1 | 3 | 3 | 2 |
| Z3-166 | 7 | 4 | 6 | 5 | 1 | 4 | 4 | 3 |
| Z3-007 | 1 | 1 | 1 | 1 | 3 | 1 | 1 | 2 |
