# Supplementary material for: Genome characterization and population genetic structure of the zoonotic pathogen, Streptococcus canis
Source: BMC Microbiol. 2012 Dec 18;12:293. doi: 10.1186/1471-2180-12-293 (PMC3541175; doi:10.1186/1471-2180-12-293)
Supplement: Additional file 6 — Ln P(D) scores for Structure analysis. [file 1471-2180-12-293-S6.doc]

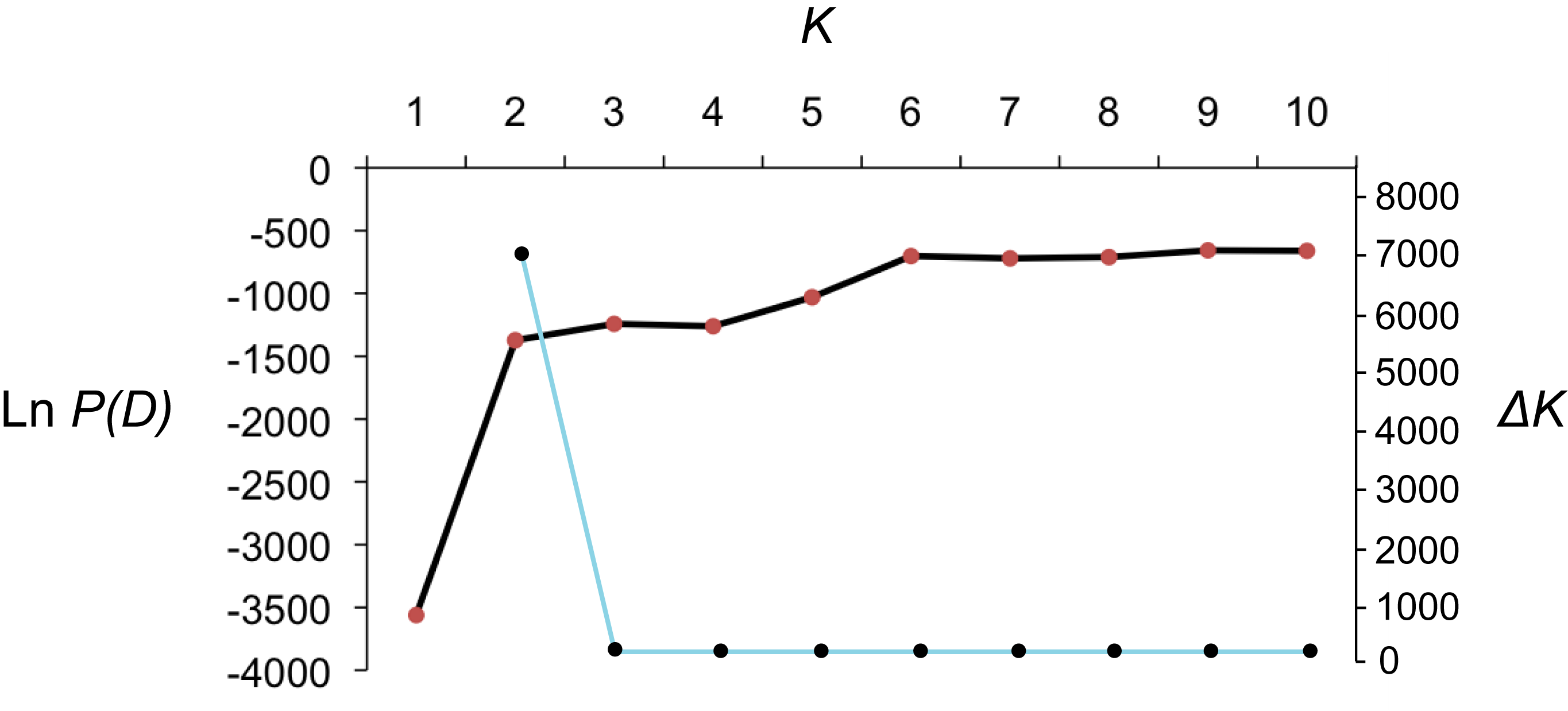


**Additional file 6.** Chart showing Ln *P(D)* scores (red dots) produced by the Structure analysis for *K*=1-10, and *∆K* values (black dots) for *K*=1-10.
