# Supplementary material for: Genome characterization and population genetic structure of the zoonotic pathogen, Streptococcus canis
Source: BMC Microbiol. 2012 Dec 18;12:293. doi: 10.1186/1471-2180-12-293 (PMC3541175; doi:10.1186/1471-2180-12-293)
Supplement: Additional file 7 — MLST PCR primer details. [file 1471-2180-12-293-S7.doc]

**Additional file 7.** MLST PCR primer details

| Locus | Primer Name | Sequence | Thermal profile |
| --- | --- | --- | --- |
| *cpn60* | RZcpnGohF (H279A)^A^ | GAIIIIGCIGGIGAYGGIACIACIAC | 94°C, 4:00; 20x (94°C, 1:00; TD^F^, 2:00; 72°C, 5:00); 20x (94°C, 1:00; 35°C, 2:00; 72°C, 5:00); 72°C, 7:00 |
|  | RZcpnGohR (H280A)^A^ | YKIYKITCICCRAAICCIGGIGCYTT |
| *gapC* | RZgapClprF^B^ | ttggtattaacggtttcggtc | 94°C, 4:00; 35x (94°C, 1:00; 50°C, 1:00; 72°C, 1:00); 72°C, 7:00 |
|  | RZgapClprR^B^ | caagttgaggagtgtaagacatttc |
| *gki* | RZgkiF (*gki*-up)^C^ | GGCATTGGAATGGGATCACC | 94°C, 4:00; 30x (94°C, 1:00; 55°C, 1:00; 72°C, 1:00); 72°C, 7:00 |
|  | RZgkiR (*gki*-down)^C^ | TCTCCCGCAGCTGACAC |
| *gtr* | RZgtrF (*gtr*-up)^D^ | GAGGTTGTGGTGATTATTGG | 94°C, 4:00; 30x (94°C, 1:00; 55°C, 1:00; 72°C, 1:00); 72°C, 7:00 |
|  | RZgtrR (*gtr*-down)^D^ | GCAAAGCCCATTTCATGAGTC |
| *mutS* | RZmutF (*mutS*-up)^D^ | GAAGAGTCATCTAGTTTAGAATACGAT | 94°C, 4:00; 30x (94°C, 1:00; 55°C, 1:00; 72°C, 1:00); 72°C, 7:00 |
|  | RZmutR (*mutS*-down)^D^ | AGAGAGTTGTCACTTGCGCGTTTGATTGCT |
| *recP* | RZrecF (*recP*-up)^D^ | GCAAATTCTGGACACCCAGG | 94°C, 4:00; 30x (94°C, 1:00; 55°C, 1:00; 72°C, 1:00); 72°C, 7:00 |
|  | RZrecR (*recP*-down)^D^ | CTTTCACAAGGATATGTTGCC |
| *tuf* | RZtufKeF (U1)^E^ | aayatgatxacxggxgcxgcxcaratgga | 94°C, 4:00; 35x (94°C, 0:30; 55°C, 0:30; 72°C, 1:00); 72°C, 7:00 |
|  | RZtufKeR (U2)^E^ | ayrttxtcxccxggcatxaccat |

^A^(Goh et al. 1997), ^B^(Zadoks et al. 2005), ^C^(Enright and Spratt 1998), ^D^(Enright et al. 2001), ^E^(Ke et al. 1999), ^F^TD = touchdown PCR from 45°C to 35°C with temperature decrease of 0.5°C per cycle.

Enright MC, Spratt BG (1998) A multilocus sequence typing scheme for Streptococcus pneumoniae: identification of clones associated with serious invasive disease. Microbiology 144 ( Pt 11):3049-3060

Enright MC, Spratt BG, Kalia A, Cross JH, Bessen DE (2001) Multilocus sequence typing of Streptococcus pyogenes and the relationships between emm type and clone. Infect Immun 69:2416-2427

Goh SH, Santucci Z, Kloos WE, Faltyn M, George CG, Driedger D, Hemmingsen SM (1997) Identification of Staphylococcus species and subspecies by the chaperonin 60 gene identification method and reverse checkerboard hybridization. J Clin Microbiol 35:3116-3121

Ke D, Picard FJ, Martineau F, Menard C, Roy PH, Ouellette M, Bergeron MG (1999) Development of a PCR assay for rapid detection of enterococci. J Clin Microbiol 37:3497-3503

Zadoks RN, Tikofsky LL, Boor KJ (2005) Ribotyping of Streptococcus uberis from a dairy's environment, bovine feces and milk. Vet Microbiol 109:257-265
